# Associations between pregnant women's attitudes toward, and literacy in, artificial intelligence, and their association with perceived influence of internet-based information on decision-making

**DOI:** 10.3389/fpubh.2026.1881831

**Published:** 2026-08-10

**Authors:** Esra Cevik

**Affiliations:** Department of Midwifery, Faculty of Health Sciences, Balikesir University, Balikesir, Türkiye

**Keywords:** AI literacy, artificial intelligence, decision-making, internet, pregnancy

## Abstract

**Objective:**

This study aimed to determine the level of perceived influence of internet-based information on decision-making among pregnant women and to examine its associations with artificial intelligence (AI) literacy and attitudes toward AI.

**Methods:**

This cross-sectional study was conducted among 423 pregnant women attending a university hospital in Balikesir, Türkiye, between March and December 2025. Data were collected using a Personal Information Form, the Decision-Making Scale via Internet on Pregnancy (DMSIP), the Artificial Intelligence Literacy Scale (AILS), and the General Attitudes toward Artificial Intelligence Scale (GAAIS). Descriptive statistics, group comparisons, correlation analyses, hierarchical multiple linear regression, structural equation modeling (SEM), and mediation analysis within the SEM framework were performed.

**Results:**

The mean DMSIP score was 34.39 ± 7.73. The hierarchical regression model explained 59.0% of the variance, with positive attitudes toward AI emerging as the strongest associated factor. In the structural equation model, the contextual latent composite construct representing selected sociodemographic and obstetric characteristics (SDS) showed the strongest direct association with DMSIP, followed by attitudes toward AI. AI literacy was not significantly associated with DMSIP in the structural model. Mediation analysis showed that attitudes toward AI mediated the association between AI literacy and DMSIP.

**Conclusion:**

The role of AI should also be considered when examining the perceived influence of internet-based information on pregnancy-related decision-making. As AI becomes increasingly integrated into pregnancy-related information environments, the development and validation of pregnancy-specific instruments to assess AI literacy and attitudes toward AI may strengthen future research in this field.

## 1 Introduction

Today, the rapid development of digital technologies is fundamentally transforming access to health-related information and decision-making processes. Pregnancy, in particular, is a critical period during which individuals are required to make continuous decisions regarding both their own health and the health of the fetus, prompting many women to seek internet-based information to support pregnancy-related decision-making (1, 2). In recent years, the widespread use of artificial intelligence (AI)-based digital health systems has further increased the complexity and multidimensional nature of accessing, interpreting, and utilizing health information in health decisions.

In Türkiye, the vast majority of women use the internet and are increasingly benefiting from digital technologies (3). However, the accuracy, reliability, and timeliness of health information available online vary considerably. This situation may increase the risk of exposure to misinformation among pregnant women and may contribute to clinically significant decision-making errors (2). The growing use of generative AI tools in the production of health information has made this issue even more critical. Although AI-based systems facilitate rapid access to health information, concerns remain regarding the accuracy, transparency, and clinical reliability of AI-generated content, particularly in pregnancy-related contexts (4). The perceived influence of internet-based information on pregnancy-related decision-making is a multidimensional phenomenon influenced by individual cognitive competencies and contextual characteristics surrounding pregnancy.

Factors such as gestational age, pregnancy intention, pregnancy complications, and socioeconomic conditions may alter women's information needs, perceived uncertainty, and perceptions of the influence of internet-based resources during pregnancy.

The Technology Acceptance Model posits that individuals' perceptions and attitudes toward a technology play a significant role in its adoption and use. According to this model, technologies perceived as useful and easy to use are more likely to be adopted by individuals (5). The eHealth Literacy model refers to individuals' ability to search for, locate, understand, evaluate, and use health information obtained from electronic sources in their health-related decisions. In this model, individuals with high levels of e-health literacy are better able to evaluate the accuracy and reliability of online health information and integrate this information into their health decisions in a more informed manner (6). Individuals with higher general AI literacy may differ in how they understand, use, and engage with AI-supported digital information environments, which may be associated with the perceived influence of internet-based information on pregnancy-related decision-making. Although previous studies have examined internet use during pregnancy (1, 7, 8), little is known about the factors associated with the perceived influence of internet-based information on pregnancy-related decision-making. Understanding these associated factors may help healthcare professionals support the safe and informed use of digital health information during pregnancy. Based on these theoretical perspectives, AI literacy, attitudes toward AI, and pregnancy-related contextual characteristics were expected to be associated with the perceived influence of internet-based information on decision-making during pregnancy.

### 1.1 Hypotheses

**H**_**1**_**:** Positive attitudes toward artificial intelligence are associated with perceived influence of internet-based information on decision-making during pregnancy.
**H**_**2**_**:** Negative attitudes toward artificial intelligence are associated with perceived influence of internet-based information on decision-making during pregnancy.
**H**_**3**_**:** Artificial intelligence literacy is associated with perceived influence of internet-based information on decision-making during pregnancy.
**H**_**4**_**:** Sociodemographic and obstetric characteristics are associated with perceived influence of internet-based information on decision-making during pregnancy.
**H**_**5**_**:** AI literacy and attitudes toward AI provide additional explanatory value beyond sociodemographic and obstetric variables in explaining perceived influence of internet-based information on decision-making during pregnancy.
**H**_**6**_**:** AI literacy, attitudes toward AI, and pregnancy-related contextual characteristics are simultaneously associated with the perceived influence of internet-based information on decision-making during pregnancy within the structural equation model.

## 2 Methods

### 2.1 Study design and participants

A cross-sectional study was conducted between March and December 2025 at Balikesir University Health Application and Research Hospital, located in Balikesir, Türkiye. Data were collected from pregnant women who visited the gynecology outpatient clinic and the maternity ward at Balikesir University Health Application and Research Hospital. This study was conducted in accordance with the Strengthening the Reporting of Observational Studies in Epidemiology (STROBE) guidelines for cross-sectional studies (9). The target population comprised pregnant women attending the gynecology outpatient clinic and maternity ward during the study period. The minimum required sample size was initially estimated as 423 using Epi Info 7.2 based on an unknown population, 50% prevalence, 95% confidence level, 5% margin of error, and 10% safety margin. Because the primary multivariable analysis was hierarchical multiple linear regression, an additional a priori power analysis was conducted using G^*^Power 3.1.9.4 (10). Assuming a medium effect size (*f* ^2^ = 0.15), α = 0.05, power = 0.95, and 14 predictors, the required sample size was 194. Therefore, the final sample of 423 participants was considered sufficient for all planned analyses. Although convenience sampling was used, the university hospital where the study was conducted is a tertiary care center that serves pregnant women from diverse socioeconomic backgrounds and various risk groups. This setting likely contributed to sufficient variability in the study variables. The inclusion criteria were; being 18 years of age or older, having visited the relevant hospital units, being able to communicate in Turkish, having no communication barriers, and voluntarily agreeing to participate in the study.

### 2.2 Measures

#### 2.2.1 Sociodemographic information form

The researcher-developed questionnaire, prepared according to the literature, collected information on participants' sociodemographic characteristics, obstetric history, and AI-related knowledge, attitudes, and behaviors. In the descriptive tables, history of miscarriage and curettage was presented as two separate variables with yes/no options. However, in multivariate analyses, because the two variables are closely related and show multicollinearity (variance inflation factor and tolerance assessments) when entered simultaneously into the regression model, they were combined under a single binary variable (history of miscarriage/curettage). Participants who previously reported a history of miscarriage or curettage were coded as “Yes,” while those who reported neither were coded as “No.” This approach was adopted to improve the stability of the model while preserving the clinical significance of previous obstetric history. For descriptive purposes, AI-related variables were categorized as knowledge about AI (adequate/inadequate), general attitude toward AI (positive/not positive), and current AI use during pregnancy (yes/no) (8, 11, 12).

#### 2.2.2 Decision making scale via internet on pregnancy (DMSIP)

The scale developed by Koyun and Erbektas (13) is a five-point Likert-type scale consisting of 10 items and two subscales (perceived self-efficacy, perceived self-control). The scale was developed to assess the extent to which information obtained from the internet is perceived to influence pregnant women's decision-making processes regarding pregnancy-related issues. The scale does not assess the frequency of internet use, trust in online information, or the objective quality of decisions. Higher scores indicate a greater perceived influence of internet information on decision-making during pregnancy. The total score ranges from 10 to 50, with higher scores indicating a greater perceived influence of internet-based information on decision-making during pregnancy (13). The Cronbach's alpha coefficient for the scale in this study is 0.93.

#### 2.2.3 AI literacy scale (AILS)

The scale developed by Wang et al. (15) and adapted into Turkish by Celebi et al. (14) consists of 12 items and four subscales (awareness, usage, evaluation, and ethics). As scores on the seven-point Likert-type scale increase, the level of AI literacy increases (14, 15). In this study, the Cronbach's alpha value was calculated as 0.81.

#### 2.2.4 General attitudes towards artificial intelligence scale (GAAIS)

Developed by Schepman and Rodway (17) and adapted into Turkish by Kaya et al. (16), this scale consists of 20 items and two subscales (positive and negative attitudes). The scale does not have a total score; the Cronbach's alpha coefficients for this study are 0.93 for positive attitude and 0.86 for negative attitude.

### 2.3 Data collection and ethical considerations

#### 2.3.1 Data collection procedure

A pilot study was conducted with 10 pregnant women to assess the comprehensibility of the data collection tools. The study evaluated whether any sentences were unclear. No substantial modifications were required following pilot testing; only minor wording revisions were made to improve clarity. After making the necessary revisions, these participants were excluded from the study. To minimize paper waste, data were collected digitally via a survey created using Google Forms. After providing participants with information about the study and obtaining informed consent, the surveys were completed via QR code on the participants' own mobile devices; for those who did not wish to use the QR code, the surveys were completed using a tablet provided by the researcher. The data collection process was completed between March 2025 and December 2025. Prior to analysis, the dataset was screened for missing values. No missing data were identified because all questionnaire items were required in the electronic survey form.

#### 2.3.2 Ethical considerations

The study was conducted in accordance with the principles of the Declaration of Helsinki. The necessary approvals for the study were obtained from the Balikesir University Health Sciences Non-Interventional Research Ethics Committee (February 13, 2024; 2024/12) and the relevant institution (July 18, 2024; E.404051). The purpose of the study was explained to the participants before data collection. Participants then provided electronic informed consent by confirming their agreement on the Google Forms consent page. Only participants who electronically agreed to participate were able to access the survey questions. The study was not preregistered in a publicly accessible registry. However, the study protocol, including the research objectives, methodology, and analysis plan, was reviewed and approved as part of the university-funded Scientific Research Projects application process prior to data collection.

#### 2.3.3 Statistical analysis

All statistical analyses were performed using IBM SPSS Statistics version 27.0 (IBM Corp., Armonk, NY, USA) and Jamovi version 2.7.23.1. Descriptive statistics are presented as mean ± standard deviation (SD) for continuous variables and frequencies with percentages for categorical variables. Normality was assessed using skewness and kurtosis (±1.5). Group comparisons were performed using Student's *t*-test, and Pearson's correlation analysis was used to examine associations between continuous variables.

To identify factors associated with perceived influence of internet-based information on decision-making during pregnancy, a theory-driven hierarchical multiple linear regression analysis was conducted. Candidate variables were selected according to theoretical relevance and bivariate associations (*p* < 0.20) (18). Variables representing AI knowledge, AI use, and general AI attitudes were not entered into the regression models because these constructs were already quantitatively represented by the validated AI literacy and attitude scales. This approach minimized conceptual overlap and improved model parsimony. Categorical variables were dummy coded and entered sequentially in four blocks: (1) sociodemographic characteristics, (2) pregnancy-related clinical and obstetric characteristics, (3) AI literacy, and (4) positive and negative attitudes toward AI. Regression assumptions were evaluated using the Durbin–Watson statistic, tolerance, variance inflation factor (VIF), residual diagnostics, and influential case statistics. Model performance was assessed using *R*^2^, adjusted *R*^2^, Δ*R*^2^, and the overall *F* statistic. Statistical significance was set at *p* < 0.05.

A confirmatory structural equation model (SEM) was subsequently performed to examine the simultaneous relationships among AI literacy, attitudes toward AI, selected sociodemographic/obstetric characteristics, and perceived influence of internet-based information on decision-making during pregnancy. The SEM comprised a measurement model and a structural model. Selected sociodemographic and obstetric characteristics were represented by a contextual latent composite construct (SDS) consisting of income level, gestational week, pregnancy complications, and pregnancy intention. These indicators were selected based on theoretical relevance and their significant contribution in the hierarchical regression analysis. Because these variables represent contextual conditions rather than manifestations of a common latent psychological construct, the SDS variable was conceptualized as a contextual latent composite. SEM parameters were estimated using the Diagonally Weighted Least Squares (DWLS) estimator because of the ordinal nature of the observed variables. Model fit was evaluated using χ^2^, Comparative Fit Index (CFI), Tucker–Lewis Index (TLI), Root Mean Square Error of Approximation (RMSEA), Standardized Root Mean Square Residual (SRMR), and Normed Fit Index (NFI). Standardized factor loadings, standardized path coefficients, and coefficients of determination (*R*^2^) were reported. An additional mediation analysis was conducted within the SEM framework to examine whether attitudes toward AI mediated the association between AI literacy and DMSIP.

## 3 Results

### 3.1 Sociodemographic, obstetric, AI related characteristics, and scale scores of participants

The mean age of the pregnant women participating in the study was 28.79 ± 5.13 years, and the mean gestational age was 21.74 ± 10.72 weeks. Most participants had at least a high school education (81.3%), reported an income equal to or greater than their expenses (92.4%), and lived in a district or provincial center (87.0%). 67.6% of the women were not employed, while the vast majority of their spouses (92.2%) were employed; 92.9% perceived their mental health status as good, and 87.5% had no chronic illnesses. The mean number of pregnancies among participants was 1.88 ± 1.06. Of the women, 17.3% reported a history of miscarriage and/or curettage, while 89.1% stated that their current pregnancy was planned. In addition, 76.6% reported experiencing no complications during pregnancy, 54.1% preferred vaginal delivery, and 51.3% indicated that their baby's gender was female. Furthermore, 77.1% of the participants reported having knowledge about artificial intelligence, 66.0% expressed a positive attitude toward artificial intelligence, and 54.4% reported using artificial intelligence during pregnancy. Among participants who used AI, ChatGPT was the most frequently reported application (64.3%). The mean DMSIP score was 34.39 ± 7.73, the mean AILS score was 56.99 ± 11.77, the mean GAAIS Positive Attitude score was 38.81 ± 9.53, and the mean GAAIS Negative Attitude score was 23.13 ± 5.56 (Table 1).

**Table 1:** Sociodemographic characteristics of pregnant women (*n* = 423).

Variables	*n* (%)/Mean ± SD
**Age**	28.79 ± 5.13
**Gestational week**	21.74 ± 10.72
Educational level	
Primary education	79 (18.7)
High school and above	344 (81.3)
Employment status	
Employed	137 (32.4)
Unemployed	286 (67.6)
Partner's employment status	
Employed	390 (92.2)
Unemployed	33 (7.8)
Income level	
Income equal to or higher than expenses	391 (92.4)
Income lower than expenses	32 (7.6)
Place of residence	
Village	55 (13.0)
District/City	368 (87.0)
Chronic disease	
Yes	53 (12.5)
No	370 (87.5)
Perceived mental health status	
Good	393 (92.9)
Poor	30 (7.1)
**Number of pregnancies**	1.88 ± 1.06
**Number of births**	0.68 ± 0.87
**Number of living children**	0.68 ± 0.84
History of miscarriage/curettage†	
Yes	73 (17.3)
No	350 (82.7)
Pregnancy complications	
Yes	99 (23.4)
No	324 (76.6)
Pregnancy intention	
Yes	377 (89.1)
No	46 (10.9)
Preferred mode of delivery	
Vaginal delivery	229 (54.1)
Cesarean section	194 (45.9)
Baby's gender (*n* = 378)	
Female	194 (51.3)
Male	184 (48.7)
Self-reported knowledge about AI	
Adequate	326 (77.1)
Inadequate	97 (22.9)
Self-reported positive attitude toward AI	
Positive	279 (66.0)
Not positive	144 (34.0)
Use of AI during pregnancy	
Yes	230 (54.4)
No	193 (45.6)
Most commonly used AI application during pregnancy (*n* = 30)	
Chat GPT	148 (64.3)
Other^*^	82 (35.7)
**DMSIP**	34.39 ± 7.73
Perceived Self-Efficacy	17.01 ± 3.79
Perceived Self-Control	17.37 ± 4.28
**Positive GAAIS**	38.81 ± 9.53
**Negative GAAIS**	23.13 ± 5.56
**AILS**	56.99 ± 11.77
Awareness	13.89 ± 3.07
Usage	13.35 ± 3.63
Evaluation	14.22 ± 4.18
Ethics	15.50 ± 3.22

**SD**, standard deviation; ^*^Gemini, Yandex AI, Microsoft Copilot. †History of miscarriage and curettage were collected as separate variables but combined into a single binary variable for multivariable analyses because of multicollinearity. **DMSIP**, decision-making scale via internet on pregnancy; **AILS**, artificial intelligence literacy scale; **GAAIS**, general attitude scale toward artificial intelligence.

### 3.2 Differences in perceived influence of internet-based information on decision-making according to participant characteristics

Table 2 summarizes DMSIP scores according to participants' sociodemographic and obstetric characteristics. Significantly higher DMSIP mean scores were observed among participants with a high school education or higher (*p* < 0.001, *d* = −0.772), those whose income was equal to or greater than their expenses (*p* < 0.001, *d* = 1.367), those who had spent most of their lives in a district or provincial center (*p* = 0.004, *d* = −0.424), those with a chronic disease (*p* < 0.001, *d* = 0.848), those without a history of miscarriage/curettage (*p* = 0.021, *d* = −0.331), those who experienced pregnancy-related complications (*p* < 0.001, *d* = 0.498), those with a planned pregnancy (*p* < 0.001, *d* = 0.774), and those who preferred vaginal birth (*p* = 0.005, *d* = 0.279). No statistically significant differences were observed according to employment status, partner's employment status, perceived mental health, or baby's gender (all *p* > 0.05).

**Table 2:** Comparison of DMSIP scores according to participants' sociodemographic and obstetric characteristics (*n* = 423).

Variables	DMSIP				
	*n*	Mean ± SD	*t*	*p*	Cohen's *d*
Educational level					
Primary education	79	29.73 ± 8.57	−5.517	**<** **0.001**	−0.772
High school and above	344	35.46 ± 7.12			
Employment status					
Employed	137	34.85 ± 8.00	0.849	0.396	0.088
Unemployed	286	34.14 ± 7.61			
Partner's employment status					
Employed	390	34.61 ± 7.59	1.758	0.087	0.366
Unemployed	33	31.78 ± 8.96			
Income level					
Income equal to or higher than expenses	391	35.14 ± 7.11	5.999	**<** **0.001**	1.367
Income lower than expenses	32	25.18 ± 9.16			
Place of residence					
Village	55	31.56 ± 8.20	−2.933	**0.004**	−0.424
District/City	368	34.81 ± 7.58			
Chronic disease					
Yes	53	39.92 ± 3.63	9.814	**<** **0.001**	0.848
No	370	33.60 ± 7.84			
Perceived mental health status					
Good	393	34.51 ± 7.71	1.145	0.253	0.217
Poor	30	32.83 ± 7.97			
History of miscarriage/curettage					
Yes	73	32.28 ± 8.62	−2.572	**0.021**	−0.331
No	350	34.83 ± 7.47			
Pregnancy complications					
Yes	99	37.28 ± 6.24	4.921	**<** **0.001**	0.498
No	324	33.50 ± 7.94			
Pregnancy intention					
Yes	377	35.02 ± 7.53	4.958	**<** **0.001**	0.774
No	46	29.19 ± 7.47			
Preferred mode of delivery					
Vaginal delivery	229	35.37 ± 7.08	2.825	**0.005**	0.279
Cesarean section	194	33.23 ± 8.31			
**Baby's gender (*****n*** = **378)**					
Female	194	35.13 ± 7.00	1.185	0.237	0.123
Male	184	34.19 ± 8.36			

Effect sizes were assessed according to Cohen's (1988) criteria. For Cohen's d, 0.20 indicates a small effect, 0.50 a moderate effect, and 0.80 a large effect.

**SD**, standard deviation, ^*^Gemini, Yandex AI, Microsoft Copilot, **DMSIP**, decision-making scale via internet on pregnancy; AILS, artificial intelligence literacy scale; **GAAIS**, general attitude scale toward artificial intelligence.

### 3.3 Correlations between continuous variables

DMSIP scores showed significant positive correlations with AI literacy, all AI literacy subscales, and both positive and negative attitudes toward AI (all *p* < 0.05; Table 3).

**Table 3:** Pearson correlations between DMSIP scores and continuous study variables (*n* = 423).

Variables	DMSIP	
	*r*	*p*
**Age**	−0.043	0.374
**Gestational week**	−0.574	**<** **0.001**
**Number of pregnancies**	−0.200	**<** **0.001**
**Number of births**	−0.191	**<** **0.001**
**Number of living children**	−0.196	**<** **0.001**
**GAAIS positive attitude**	0.619	**<** **0.001**
**GAAIS negative attitude**	0.130	**0.007**
**AILS**	0.474	**<** **0.001**
Awareness	0.402	**<** **0.001**
Usage	0.438	**<** **0.001**
Evaluation	0.456	**<** **0.001**
Ethics	0.263	**<** **0.001**

**r:** Pearson's correlation coefficient; **DMSIP**, decision-making scale via internet on pregnancy; **AILS**, artificial intelligence literacy scale; **GAAIS**, general attitude toward artificial intelligence scale.

### 3.4 Predictors of perceived influence of internet-based information on decision-making during pregnancy (hierarchical regression analysis)

Regression assumptions were satisfied. Residual independence (Durbin–Watson = 1.931), multicollinearity diagnostics (tolerance = 0.512–0.913; VIF = 1.095–1.955), residual plots, and influential case statistics indicated no major violations of model assumptions. Visual inspection of the histogram, Normal P–P plot, and scatterplot of standardized residuals supported the assumptions of normality, linearity, and homoscedasticity. In addition, Cook's distance, leverage values, Mahalanobis distance, and studentized deleted residuals indicated no influential outliers. Therefore, all assumptions required for hierarchical multiple linear regression were considered to be satisfied.

To evaluate the relative contributions of factors associated with the perceived influence of internet-based information on decision-making during pregnancy, variables were entered into a hierarchical regression model in four blocks based on a theoretical framework. In the first block, educational level, spouse's employment status, income level, place of residence, and chronic disease status were included in the model. These variables represent socioeconomic and structural characteristics that were evaluated as key control variables potentially associated with access to health information, use of digital resources, and the perceived influence of internet-based information on decision-making. In the second block, history of miscarriage, pregnancy complications, planned pregnancy, preferred mode of delivery, gestational week, and number of pregnancies were added to the model. These variables represent clinical and obstetric characteristics specific to pregnancy and were included based on the assumption that pregnancy-related risk and experience may be associated with information-seeking needs and the perceived influence of internet-based information on decision-making. In the third block, the Artificial Intelligence Literacy Scale was entered into the model. This block aimed to determine the unique contribution of individuals' competencies in understanding, evaluating, and using AI technologies to the perceived influence of internet-based information on pregnancy-related decision-making. In the final block, Positive Attitudes Toward Artificial Intelligence and Negative Attitudes Toward Artificial Intelligence were added to the model. The primary rationale for entering these variables in the final step was based on the Technology Acceptance Model and related technology acceptance theories, which suggest that cognitive evaluations of technology may provide additional explanatory value beyond technology literacy alone (5).

The results of the four-step hierarchical regression analysis conducted to identify variables associated with the perceived influence of internet-based information on decision-making are presented in Table 4. In the first model, sociodemographic variables explained 21.1% of the variance in perceived influence of internet-based information on decision-making scores (*R*^2^ = 0.211, Adjusted *R*^2^ = 0.201, *F*(5,417) = 22.271, *p* < 0.001). The addition of pregnancy-specific clinical and obstetric variables in the second model significantly increased the explanatory power of the model, raising the explained variance to 44.9% (Δ*R*^2^ = 0.238, *p* < 0.001). In the third step, the Artificial Intelligence Literacy Scale was added to the model and made a significant additional contribution (Δ*R*^2^ = 0.057, *p* < 0.001). In the final step, the inclusion of positive and negative attitudes toward artificial intelligence increased the explained variance to 59.0% (*R*^2^ = 0.590, Adjusted *R*^2^ = 0.576), and attitudinal variables provided an additional 8.4% explanatory contribution to the model (Δ*R*^2^ = 0.084, *p* < 0.001).

**Table 4:** Hierarchical regression analysis of factors associated with perceived influence of internet-based information on decision-making during pregnancy (DMSIP) (*n* = 423).

Model fit statistics	Variables entered	*R* ^2^	Adj. *R*^2^	Δ*R*^2^	*F* (df)		
1	Sociodemographic variables	0.211	0.201	–	22.271 (5,417)^***^		
2	+ Pregnancy-related clinical and obstetric variables	0.449	0.434	0.238	30.409 (11,411)^***^		
3	+ AI literacy	0.506	0.492	0.057	35.011 (12,410)^***^		
4	+ Attitudes toward AI	0.590	0.576	0.084	41.879 (14,408)^***^		
**Variables**	***B***	**SE**	β	***p***	**95% CI**	**Tol**.	**VIF**
Regression coefficients (final model–model 4)							
**Constant**	17.778	2.637	–	<0.001	12.594–22.961	–	–
**Education level**	0.581	0.740	0.029	0.433	−0.874–2.036	0.722	1.385
**Partner employment**	−0.168	1.049	−0.006	0.873	−2.231–1.894	0.759	1.318
**Income level**	4.206	1.125	0.144	**<0.001**	1.995–6.417	0.679	1.472
**Place of residence**	−0.142	0.768	−0.006	0.854	−1.652–1.368	0.900	1.111
**Chronic disease**	1.295	0.795	0.055	0.104	−0.267–2.857	0.868	1.152
**History of miscarriage/curettage**	−0.179	0.809	−0.009	0.825	−1.770–1.411	0.642	1.557
**Pregnancy complications**	1.922	0.635	0.105	**0.003**	0.674–3.170	0.832	1.202
**Pregnancy intention**	2.118	0.863	0.085	**0.015**	0.422–3.815	0.833	1.201
**Preferred mode of delivery**	0.708	0.521	0.046	0.175	−0.316–1.732	0.892	1.121
**Gestational week**	−0.237	0.026	−0.329	**<0.001**	−0.288-−0.187	0.790	1.266
**Number of pregnancies**	−0.025	0.323	−0.003	0.940	−0.660–0.611	0.512	1.955
**AI Literacy Scale (AILS)**	0.079	0.026	0.120	**0.002**	0.028–0.129	0.660	1.516
**Positive attitude toward AI**	0.302	0.033	0.372	**<0.001**	0.237–0.367	0.603	1.659
**Negative attitude toward AI**	−0.052	0.046	−0.037	0.261	−0.143–0.039	0.913	1.095

***B* =** unstandardized regression coefficient; **SE** **=** standard error; **β** **=** standardized regression coefficient; **CI** **=** confidence interval; **Tol**. **=** tolerance; **VIF** **=** variance inflation factor. The final model explained 59.0% of the variance in the perceived influence of internet-based information on decision-making during pregnancy (*R*^2^ = 0.590; adjusted *R*^2^ = 0.576), F(14, 408) = 41.879, *p* < 0.001. The increase in explained variance was significant after each block was entered (Δ*R*^2^ = 0.238, 0.057 and 0.084, respectively). The Durbin–Watson statistic (1.931) indicated no autocorrelation among residuals. Tolerance values ranged from 0.512 to 0.913, whereas VIF values ranged from 1.095 to 1.955, indicating no multicollinearity. Examination of standardized residuals, histogram, Normal P–P plot, and scatterplot of standardized residuals against standardized predicted values supported the assumptions of linearity, normality, homoscedasticity, and independence of errors. Cook's distance, leverage values, and Mahalanobis distance indicated no influential outliers.

**Dummy coding:** education level (0 = primary school, 1 = high school or above); partner employment (0 = unemployed, 1 = employed); income level (0 = income lower than expenses, 1 = income equal to or higher than expenses); place of residence (0 = village, 1 = town/city); chronic disease (0 = no, 1 = yes); history of miscarriage / curettage (0 = neither miscarriage nor curettage, 1 = history of miscarriage/curettage); pregnancy complications (0 = no, 1 = yes); pregnancy intention (0 = unplanned, 1 = planned); preferred mode of delivery (0 = cesarean section, 1 = vaginal delivery).

According to the final model of the four-step hierarchical regression analysis, the strongest associated factor of perceived influence of internet-based information on decision-making was positive attitudes toward artificial intelligence (β = 0.372, *p* < 0.001). This was followed by gestational week (β = −0.329, *p* < 0.001), income level (β = 0.144, *p* < 0.001), AI literacy (β = 0.120, *p* = 0.002), experiencing pregnancy complications (β = 0.105, *p* = 0.003), and planned pregnancy (β = 0.085, *p* = 0.015).

### 3.5 Structural equation modeling and mediation analysis

The SEM comprised three psychometric latent constructs (AI literacy, attitudes toward AI, and DMSIP) together with one contextual latent composite construct (SDS) representing selected sociodemographic and obstetric characteristics. The SDS construct was indicated by income level, gestational week, pregnancy complications, and pregnancy intention. The model was tested on 423 participants using the DWLS method. The model converged with 39 free parameters. In the goodness-of-fit assessment, the model showed an acceptable/good level of fit when scaled values were considered: scaled χ^2^(48) = 84.1, *p* < 0.001; RMSEA = 0.042, 95% CI [0.027–0.057]; SRMR = 0.053; CFI = 0.961; TLI = 0.946. In addition, GFI = 0.999 and AGFI = 0.999 were found. The model explained a significant proportion of the variance in the perceived influence of internet-based information on decision-making during pregnancy (DMSIP *R*^2^ = 0.824).

The SDS construct was represented by four observed indicators, with income level and gestational week showing the strongest standardized loadings.

According to the structural model results, the strongest standardized association with DMSIP came from sociodemographic and pregnancy-related characteristics (SDS → DMSIP: β = 0.734, *p* < 0.001). The association of general attitude towards artificial intelligence on DMSIP is also positive and significant (GAAIS → DMSIP: β = 0.296, *p* = 0.007). In contrast, the direct effect of AI literacy is not significant (AILS → DMSIP: β = −0.046, *p* = 0.545).

To further clarify the inconsistent findings for AI literacy across the hierarchical regression and SEM, an additional mediation model was tested within the SEM framework. AI literacy was significantly associated with attitudes toward AI (β = 0.458, *p* < 0.001), and attitudes toward AI was significantly associated with DMSIP (β = 0.296, *p* = 0.007). The direct effect of AI literacy on DMSIP was not significant (β = −0.046, *p* = 0.545). However, the indirect effect of AI literacy on DMSIP through attitudes toward AI was significant (B = 0.187, SE = 0.079, 95% CI [.032,0.341], β = 0.135, *p* = 0.018). The total effect of AI literacy on DMSIP was not significant (β = 0.089, *p* = 0.289). These findings indicate that AI literacy was indirectly associated with DMSIP through attitudes toward AI when the constructs were modeled at the latent level (Table 5).

**Table 5:** SEM-based mediation analysis testing attitudes toward AI as a mediator between AI literacy and DMSIP.

Path/Parameter	B	SE	95% CI for B	β	*z*	*P*
Structural paths						
**GAAIS** **←** **AILS**	1.636	0.277	1.093 to 2.178	0.458	5.911	<0.001
**GAAIS** **←** **SDS**	4.382	1.384	1.670 to 7.095	0.328	3.166	0.002
**DMSIP** **←** **AILS**	−0.063	0.105	−0.269 to 0.142	−0.046	−0.606	0.545
**DMSIP** **←** **GAAIS**	0.114	0.042	0.031 to 0.197	0.296	2.692	0.007
**DMSIP** **←** **SDS**	3.780	0.917	1.984 to 5.577	0.734	4.124	<0.001
Covariance						
**AILS** **↔** **SDS**	0.833	0.162	0.516 to 1.150	0.531	5.15	<0.001
Defined effects						
**Indirect effect: AILS** **→** **GAAIS** **→** **DMSIP**	0.187	0.079	0.032 to 0.341	0.135	2.370	0.018
**Total effect: AILS** **→** **DMSIP**	0.123	0.116	−0.104 to 0.351	0.089	1.061	0.289

**AILS** **=** artificial intelligence literacy scale; GAAIS, general attitudes toward artificial intelligence scale; DMSIP, decision-making scale via internet on pregnancy; SDS, contextual sociodemographic and pregnancy-related construct. B, unstandardized coefficient; SE, standard error; β, standardized coefficient; CI, confidence interval. The mediation model was estimated using DWLS with robust standard errors. Model fit was acceptable/good based on scaled fit indices: scaled χ^2^(48) = 84.1, *p* < 0.001; RMSEA = 0.042, 95% CI [0.027, 0.057]; SRMR = 0.053; CFI = 0.961; TLI = 0.946.

**Figure 1:**
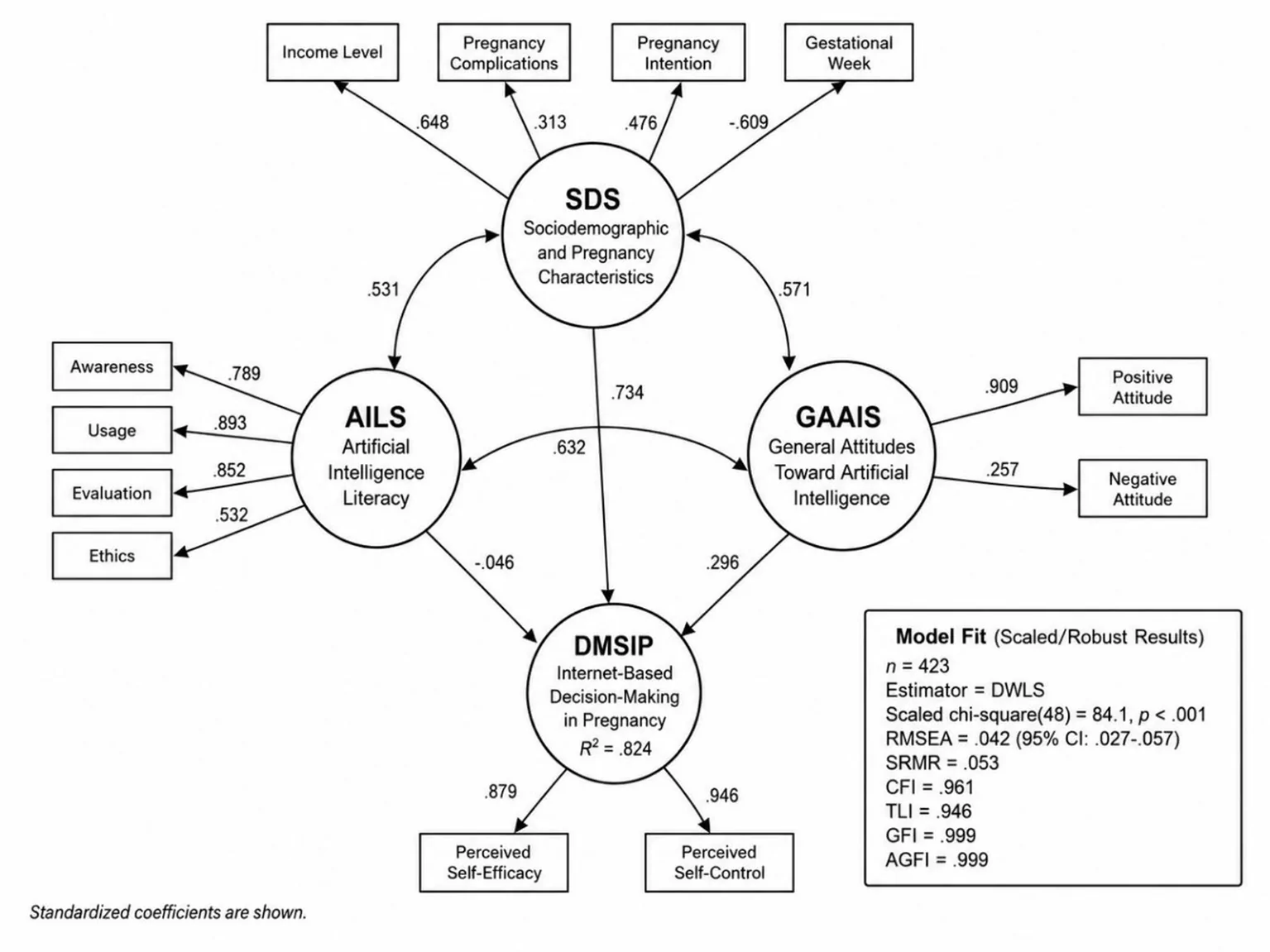
Structural equation model showing the relationships among the contextual sociodemographic/obstetric construct (SDS), artificial intelligence literacy, attitudes toward artificial intelligence, and perceived influence of internet-based information on decision-making during pregnancy.

The measurement model showed that DMSIP is strongly represented by two sub-dimensions: Perceived Self-Control is the strongest indicator (β = 0.946, *p* < 0.001), while Perceived Self-Efficacy also has a high factor loading (β = 0.879). In the AILS structure, the strongest indicators were Usage and Evaluation, while in the GAAIS structure, it was Positive Attitude. In the SDS structure, income level and gestational week stand out as stronger indicators, while pregnancy complications gave a lower but significant loading (Figure 1).

## 4 Discussion

This study found that the perceived influence of internet-based information on decision-making during pregnancy was at a moderate-to-high level. This finding is consistent with previous studies indicating that internet-based information plays an important role in the perception of pregnancy-related decision-making (1, 19, 20). However, previous studies conducted in Türkiye have reported lower mean DMSIP scores among pregnant women with lower educational attainment (21, 22). In the present study, most participants had attained at least a high school education and reported that their household income was equal to or greater than their expenses. According to the Turkish Statistical Institute's (TurkStat) 2025 Artificial Intelligence Statistics, the use of generative AI increases with educational attainment. The rate of AI use was reported as 36.1% among individuals with higher education, 22.8% among high school graduates, 17.2% among primary/secondary school graduates, and 2.2% among primary school graduates (23). Accordingly, the findings of the present study should not be generalized to pregnant women with lower educational attainment, lower socioeconomic status, or limited access to digital technologies.

Among all variables examined, positive attitudes toward artificial intelligence (AI) showed the strongest association with the perceived influence of internet-based information on decision-making during pregnancy. This finding can be interpreted within the framework of the Technology Acceptance Model, which proposes that individuals are more likely to adopt and use a technology when they perceive it as useful and relevant to their needs (5). In the context of pregnancy, women with more favorable attitudes toward AI may perceive AI-supported and internet-based information sources as having a greater influence when seeking guidance on pregnancy-related issues (24). A previous study reported an association between attitudes toward technology, digital literacy, and digital health decision-making among pregnant women (25). Similarly, general AI literacy has been conceptualized as a competency that enables individuals to critically evaluate AI technologies and engage with them effectively across different contexts (26). Gestational age was also associated with the perceived influence of internet-based information on pregnancy-related decision-making. Women in early pregnancy may experience greater uncertainty regarding pregnancy symptoms, fetal development, and lifestyle modifications, increasing their need for information and support from online sources (27). As pregnancy progresses, accumulated experience and more frequent contact with healthcare professionals may reduce the perceived influence of internet-based information on pregnancy-related decision-making.

Furthermore, higher income, experiencing pregnancy complications, and having a planned pregnancy were positively associated with the perceived influence of internet-based information on decision-making. These findings may reflect greater access to health information, heightened risk perception, and more active engagement in pregnancy care, which may contribute to increased use of digital health resources (27–30). In contrast, educational level, place of residence, and negative attitudes toward AI were not significant predictors, suggesting that the perceived influence of internet-based information on decision-making is shaped less by sociodemographic characteristics than by cognitive and attitudinal factors related to technology (6, 13). The lack of an independent association with educational level may reflect the relatively homogeneous educational profile of the study sample, in which most participants had attained at least a high school education. Similarly, negative attitudes toward AI may not necessarily reduce the perceived influence of internet-based information on pregnancy-related decision-making, particularly when digital resources have become an integral part of routine pregnancy information seeking. Collectively, these findings indicate that the successful implementation of AI-supported digital health interventions for pregnant women requires not only adequate technological infrastructure but also educational initiatives that enhance AI literacy and user-centered strategies that foster trust in AI technologies.

The mediation analysis suggests that the association between AI literacy and the perceived influence of internet-based information on pregnancy-related decision-making operates primarily through attitudes toward AI rather than through a direct pathway. This finding is consistent with the conceptualization of AI literacy as a multidimensional competency that extends beyond technical knowledge and enables individuals to critically evaluate and effectively engage with AI technologies (26). Likewise, the concept of eHealth literacy suggests that possessing the skills to obtain, understand, evaluate, and apply health information does not necessarily translate directly into health-related decision-making behaviors (6). These findings are also consistent with technology acceptance perspectives, which emphasize attitudes as more proximal determinants of technology-related behaviors.

The increasing use of internet-based and AI-generated information during pregnancy also raises important concerns regarding misinformation, information reliability, privacy, and the need for professional guidance. Although digital technologies facilitate rapid access to pregnancy-related information, the quality and accuracy of online information remain highly variable (31). Consequently, both pregnant women and healthcare professionals may encounter misinformation, incomplete information, and uncertainty within the contemporary digital information environment. In addition, the World Health Organization has highlighted the importance of privacy, transparency, accountability, and human oversight in the application of AI in healthcare (4). Therefore, midwives, nurses, and other maternal healthcare professionals should routinely assess pregnant women's use of online and AI-based information sources, guide them toward reliable evidence-based resources, and strengthen their capacity to critically appraise digital health information.

### 4.1 Limitations and strengths

Several limitations should be considered when interpreting the findings. First, this study was conducted at a single tertiary university hospital using convenience sampling, and most participants had relatively high educational attainment and adequate income, which may limit the generalizability of the findings. The DMSIP assesses pregnant women's perceived influence of internet-based information on decision-making rather than actual decision-making behaviors, objective reliance on internet-based information, or the quality of the decisions made. Finally, because the data were based on self-report questionnaires and the study employed a cross-sectional design, reporting bias may have occurred and the observed associations should not be interpreted as causal. In addition, because AI technologies evolve rapidly, women's knowledge, attitudes, and patterns of AI use may change over time. Therefore, the findings should be interpreted within the technological context in which the study was conducted.

## 5 Conclusions

The findings indicate that the perceived influence of internet-based information on pregnancy-related decision-making is more strongly associated with positive attitudes toward artificial intelligence and contextual pregnancy characteristics than with AI literacy alone. The supplementary SEM-based mediation analysis further showed that AI literacy was indirectly associated with DMSIP through attitudes toward AI, indicating that AI literacy may support pregnancy-related perceived influence of internet-based information on decision-making primarily by fostering more favorable attitudes toward AI rather than through an independent direct pathway.

## Data Availability

The datasets presented in this article are not readily available. If necessary, the data set can be shared. Requests to access the datasets should be directed to esra.cevik@balikesir.edu.tr.
